# Temperature-dependent solid-state phase transition with twinning in the crystal structure of 4-meth­oxy­anilinium chloride

**DOI:** 10.1107/S2056989023010812

**Published:** 2024-01-01

**Authors:** Rao M. Uppu, Sainath Babu, Frank R. Fronczek

**Affiliations:** a Department of Environmental Toxicology, Southern University and A&M College, Baton Rouge, LA 70813, USA; b Department of Chemistry, Louisiana State University, Baton Rouge, LA 70803, USA; University of Aberdeen, United Kingdom

**Keywords:** crystal structure, 4-alk­oxy­acetanilides, 4-alk­oxy­anilinium salts, nonsteroidal analgesics, mechanisms of toxicity

## Abstract

At room temperature, the title salt, C_7_H_10_NO^+^·Cl^−^, is ortho­rhom­bic, space group *Pbca* with *Z*′ = 1, as previously reported [Zhao (2009 ▸). *Acta Cryst.* E**65**, o2378]. Between 250 and 200 K, there is a solid-state phase transition to a twinned monoclinic *P*2_1_/*c* structure with *Z*′ = 2. We report the high temperature structure at 250 K and the low-temperature structure at 100 K. In the low-temperature structure, the –NH_3_ hydrogen atoms are ordered and this group has a different orientation in each independent mol­ecule, in keeping with optimizing N—H⋯Cl hydrogen bonding, some of which are bifurcated: these hydrogen bonds have N⋯Cl distances in the range 3.1201 (8)–3.4047 (8) Å. In the single cation of the high-temperature structure, the NH hydrogen atoms are disordered into the average of the two low-temperature positions and the N⋯Cl hydrogen bond distances are in the range 3.1570 (15)–3.3323 (18) Å. At both temperatures, the meth­oxy group is nearly coplanar with the rest of the mol­ecule, with the C—C—O—C torsion angles being −7.0 (2)° at 250 K and −6.94 (12) and −9.35 (12)° at 100 K. In the extended ortho­rhom­bic structure, (001) hydrogen-bonded sheets occur; in the monoclinic structure, the sheets propagate in the (010) plane.

## Abstract 2

The room temperature (298 K) structure of the title salt has been redetermined with disordered H atoms for the –NH_3_ group. Additionally, a twinned monoclinic structure has been identified with lower symmetry at low temperature (100 K) in which the H atoms of the –NH_3_ groups are ordered to optimize N—H⋯Cl hydrogen bonding.

## 1. Chemical context

4-Alk­oxy­acetanilides (4-AAs), represented by phenacetin, or *N*-(4-eth­oxy­phen­yl)acetamide (C_10_H_13_NO_2_), played a pivotal role in introducing synthetic fever reduction and non-opioid analgesics to the global pharmaceutical market in the early 1900s. The analgesic effects of 4-AAs result from their impact on the sensory tracts of the spinal cord, while their anti­pyretic actions predominantly occur in the brain, where they lower the temperature set point (Dalmann *et al.*, 2015 ▸; Flower & Vane, 1972 ▸). *In vivo*, the primary metabolic pathway involves oxidative O-de­alkyl­ation, producing *N*-(4-hy­droxy­phen­yl)acetamide, C_8_H_9_NO_3_, a clinically significant analgesic (Nohmi *et al.*, 1984 ▸). However, a minor fraction may undergo de­acyl­ation, leading to the formation of carcinogenic and kidney-damaging 4-alk­oxy­anilines Nohmi *et al.*, 1984 ▸; Prescott, 1980 ▸). This study centers on the crystal structure analysis of the title salt, 4-meth­oxy­aniline hydro­chloride (4-meth­oxy­anilinium chloride or 4-MAC), **I**, aiming to expound not only its potential kidney-damaging properties but also provide structural data for exploring mol­ecular targets through mol­ecular docking and mol­ecular dynamic simulations. 
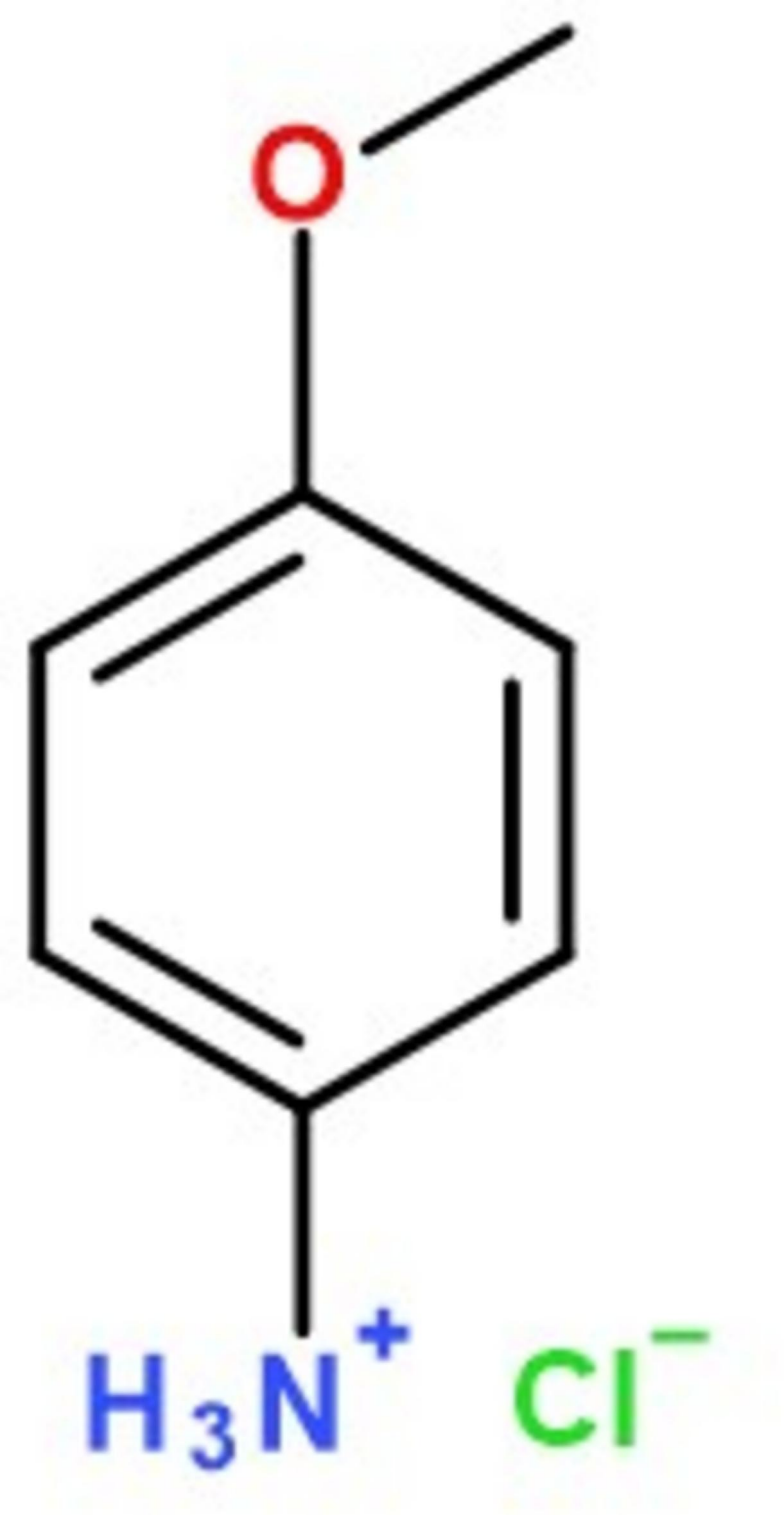


## 2. Structural commentary

At room temperature (298 K), **I** crystallizes in the ortho­rhom­bic space group *Pbca* with one formula unit in the asymmetric unit. The cell dimensions are *a* = 8.8778 (5), *b* = 8.4660 (5), *c* = 21.7236 (11) Å and *V* = 1632.73 (16) Å^3^. Cooling the sample causes a solid-state phase transition to a twinned structure with lower symmetry and two formula units in the asymmetric unit. At 250 K, the structure is still ortho­rhom­bic, but at 200 K, the space group is monoclinic *P*2_1_/*c* with *a* = 8.3772 (11), *b* = 21.715 (3), *c* = 8.8466 (12) Å, β = 90.039 (4)° and *V* = 1609.3 (4) Å^3^. At *T* = 100 K, the monoclinic cell parameters are *a* = 8.3039 (6), *b* = 21.6993 (15), *c* = 8.8495 (6) Å, β = 90.077 (2)° and *V* = 1594.58 (19) Å^3^.

**Figure 1:**
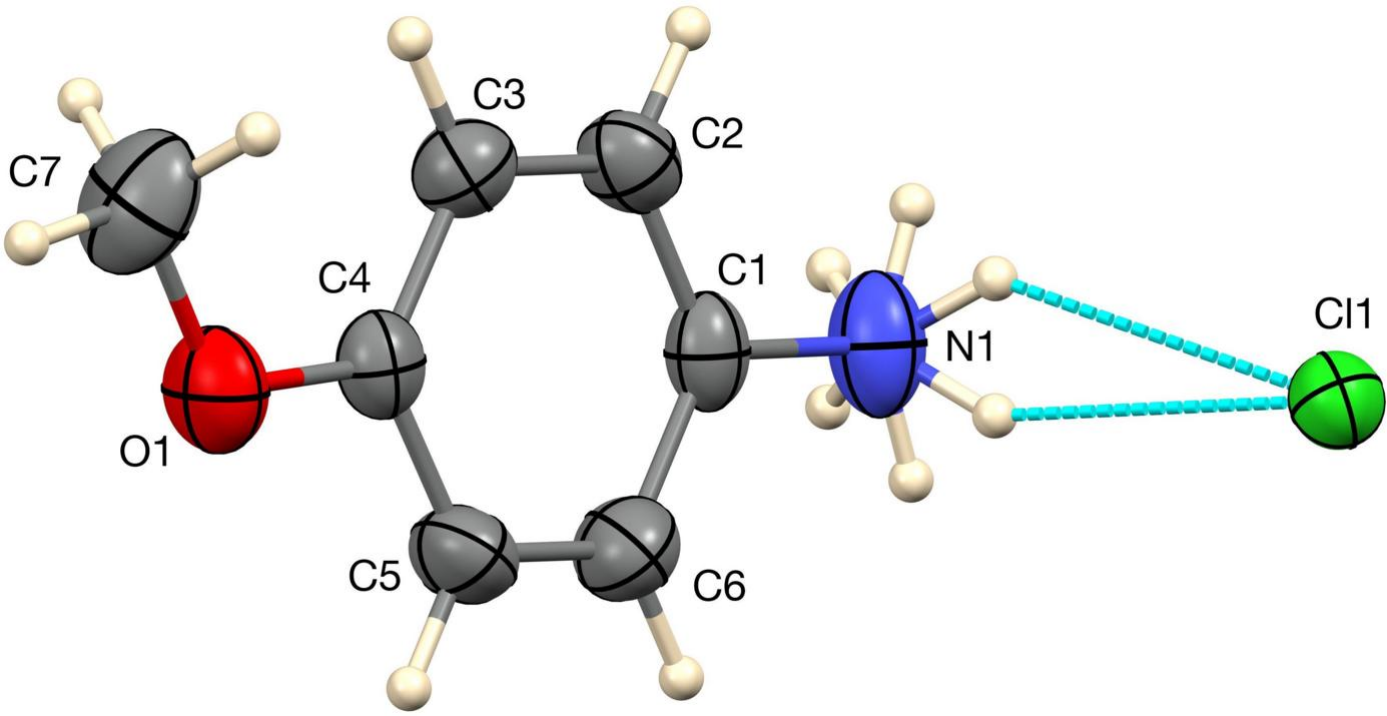
The asymmetric unit of the 250 K structure of **I**, with 50% displacement ellipsoids and hydrogen bonds indicated by blue dashed lines.

The crystal structure at 250 K, shown in Fig. 1 ▸, closely aligns with the published structure (Zhao, 2009 ▸) except that we find disorder in the NH hydrogen atoms, while Zhao treated them as ordered. Using Zhao’s intensity data, we do see evidence of a second orientation of the NH_3_ group, and we also see it from our crystal when warmed to 298 K. The meth­oxy group in **I** is nearly coplanar with the rest of the mol­ecule, with the torsion angle C7—O1—C4—C3 = −7.0 (2)°.

**Figure 2:**
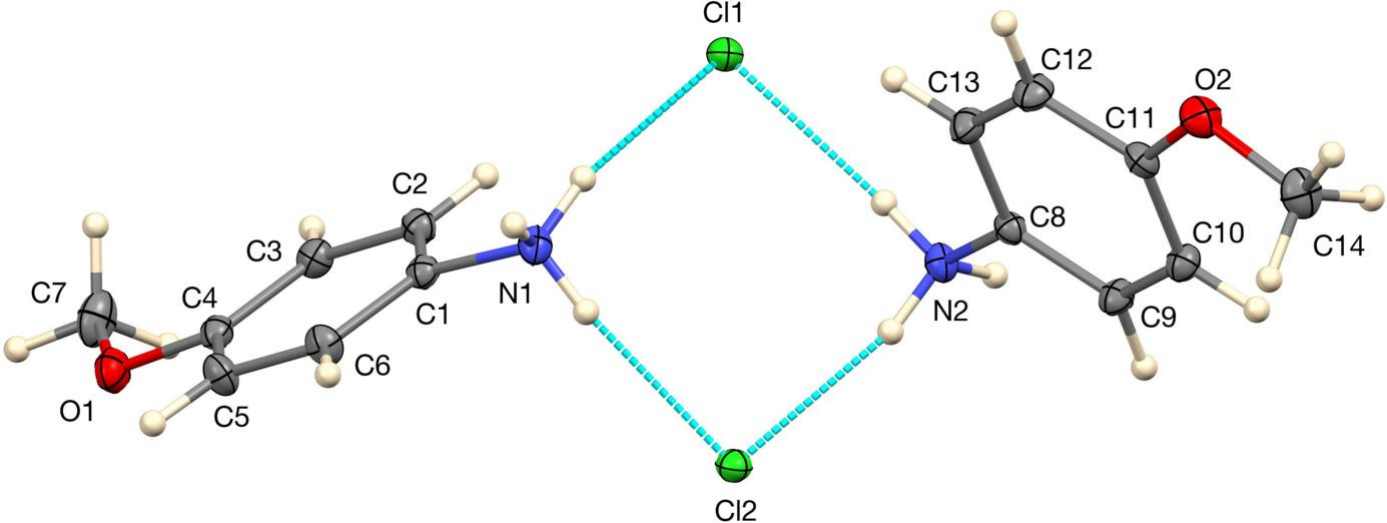
The asymmetric unit of the 100 K structure of **I**, with 50% displacement ellipsoids and hydrogen bonds indicated by blue dashed lines.

The asymmetric unit of the 100 K structure is shown in Fig. 2 ▸. The major difference between the two independent mol­ecules is the conformation of the –NH_3_ group, in which one mol­ecule has one set of the disordered positions in the 250 K structure and the second has the other. As in the 250 K structure, the meth­oxy groups are twisted only slightly out of the planes of the aromatic rings, with C7—O1—C4—C3 and C14—O2—C11—C10 torsion angles of −6.94 (12) and −9.35 (12)°, respectively.

## 3. Supra­molecular features

**Figure 3:**
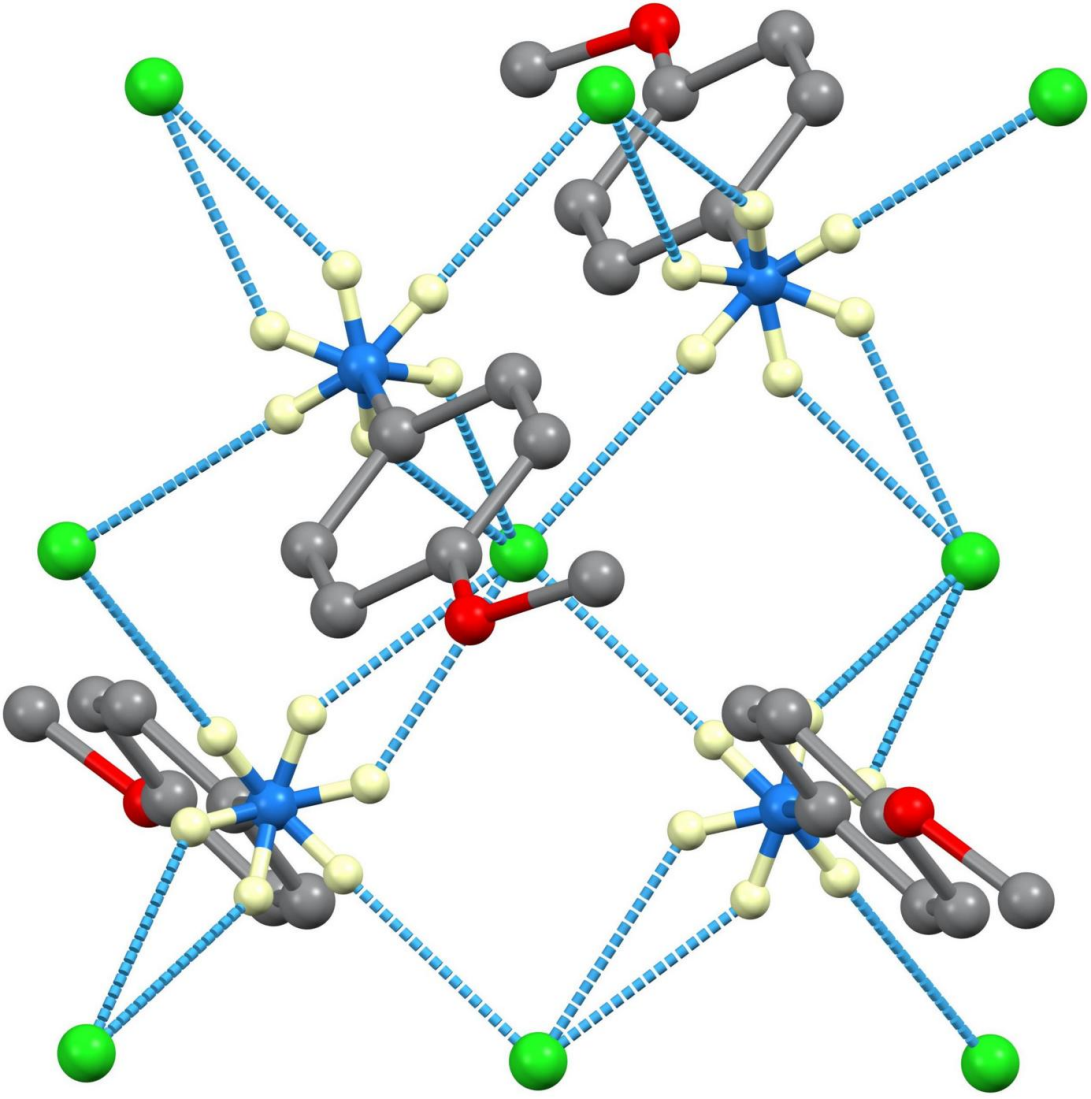
Hydrogen bonding in the 250 K structure of **I**.

**Figure 4:**
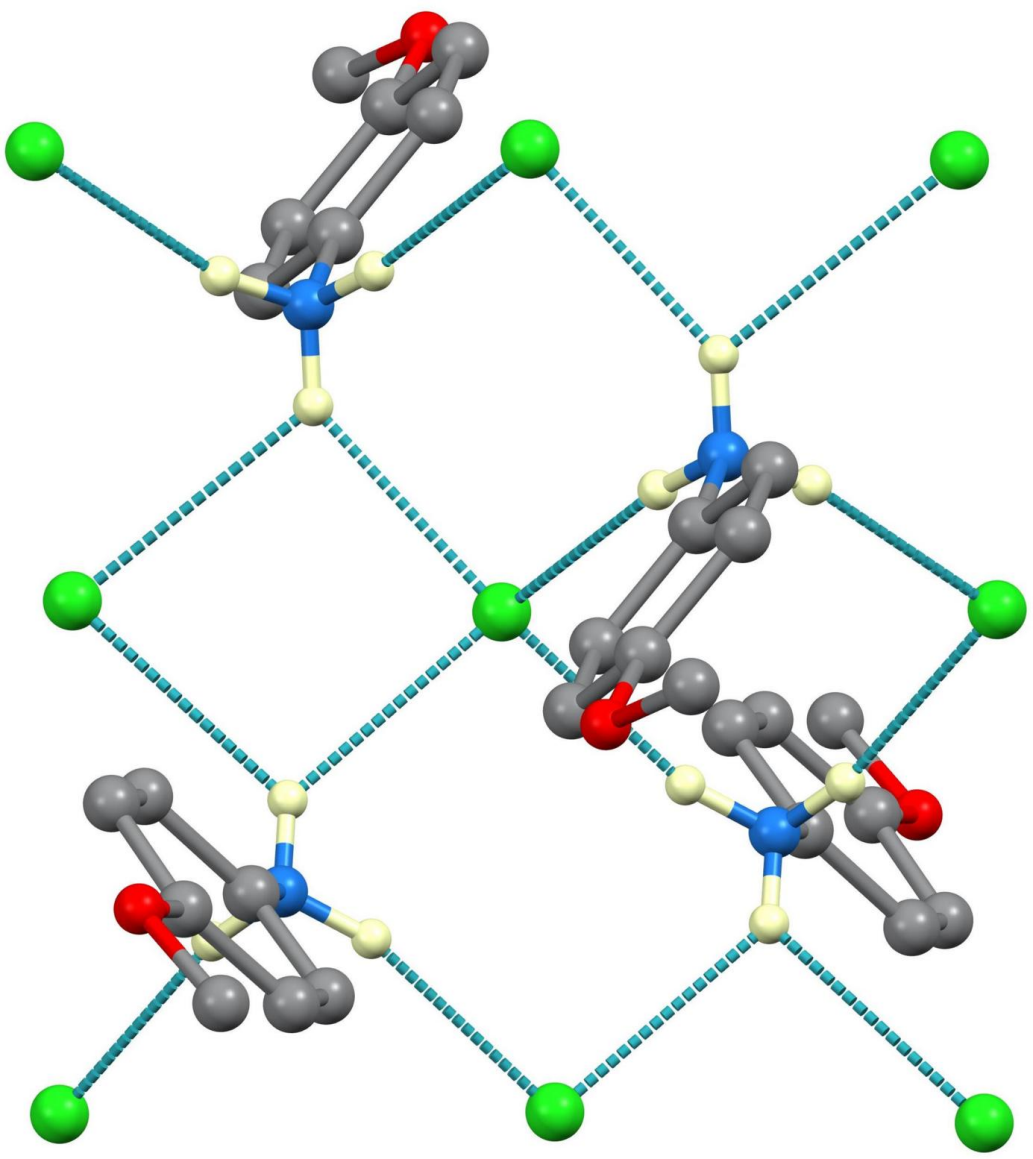
Hydrogen bonding in the 100 K structure of **I**.

In both structures, the inter­molecular inter­actions are predominantly N—H⋯Cl hydrogen bonds, as listed in Tables 1 ▸ and 2 ▸ and illustrated in Figs. 3 ▸ and 4 ▸. The N⋯Cl separations are in the range 3.1201 (8)–3.4047 (8) Å in the monoclinic 100 K structure and 3.1570 (15)–3.3323 (18) Å in the ortho­rhom­bic 250 K structure. In Fig. 4 ▸, it can be seen that for each NH_3_ group, two of the H atoms are involved in direct hydrogen bonds and the third in a bifurcated N—H⋯(Cl,Cl) bond with two acceptors. The graph set (Etter *et al.*, 1990 ▸) patterns are centrosymmetric 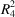 (8) rings and 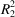 (4) rings. At 250 K, (001) sheets arise in the extended structure and at 100 K similar sheets propagate in the (010) plane, due to the change in unit-cell settings.

## 4. Database survey

A review of the literature revealed that the room temperature (298 K) structure of 4-meth­oxy­anilinium chloride was previously reported (Zhao, 2009 ▸; Cambridge Structural Database refcode CUCTUQ). Similarly, the structure of 4-eth­oxy­anilinium chloride at 100 K has been documented (Fu, 2010 ▸; Hines *et al.*, 2023 ▸). However, these studies did not provide information on phase transitions and twinning.

## 5. Synthesis and crystallization

A saturated solution of the title compound, C_7_H_10_ClNO (CAS 20265-97-8 from AmBeed, Arlington Heights, IL, USA) in boiling water was allowed to pass through a short column of activated charcoal. The resulting colorless solution (eluent) was left to cool to room temperature and evaporate slowly in the dark. Pink laths of **I**, prepared through this process, were suitable for X-ray diffraction studies.

## 6. Refinement

Crystal data, data collection and structure refinement details are summarized in Table 3 ▸. For the structure at 250 K, all the H atoms were located in difference maps and those on carbon were relocated to geometrically idealized positions with C—H = 0.94 Å and *U*_iso_(H) = 1.2*U*_eq_(C) for the aromatic C atoms and C—H = 0.97 Å and *U*_iso_(H) = 1.5*U*_eq_(C) for the methyl group. The N-bound H atoms were idealized as six half-populated sites at 60° torsional inter­vals with N—H = 0.90 Å and *U*_iso_(H) = 1.5*U*_eq_(N) and the torsion angle was refined. For the structure at 100 K, the H atoms were handled similarly, except that C—H distances were fixed at 0.95 Å for aromatic C atoms and 0.98 Å for the methyl group, and the H atoms on N were ordered with their positions individually refined. The twin law for the monoclinic structure is (1 0 0, 0 −1 0, 0 0 −1) and the BASF parameter refined to 0.4484 (6).

## Supplementary Material

Crystal structure: contains datablock(s) I_250K, I_100K, global. DOI: 10.1107/S2056989023010812/hb8089sup1.cif

Structure factors: contains datablock(s) I_250K. DOI: 10.1107/S2056989023010812/hb8089I_250Ksup2.hkl

Structure factors: contains datablock(s) I_100K. DOI: 10.1107/S2056989023010812/hb8089I_100Ksup3.hkl

CCDC references: 2314991, 2314990

Additional supporting information:  crystallographic information; 3D view; checkCIF report

## Figures and Tables

**Table 1:** Hydrogen-bond geometry (Å, °) for **I** at 250 K[Chem scheme1]

*D*—H⋯*A*	*D*—H	H⋯*A*	*D*⋯*A*	*D*—H⋯*A*
N1—H1*N*⋯Cl1^i^	0.90	2.43	3.3265 (18)	174
N1—H2*N*⋯Cl1^ii^	0.90	2.40	3.1915 (15)	147
N1—H3*N*⋯CL1	0.90	2.45	3.1570 (16)	135
N1—H4*N*⋯Cl1^iii^	0.90	2.43	3.3323 (18)	180
N1—H5*N*⋯Cl1	0.90	2.36	3.1570 (15)	148
N1—H6*N*⋯Cl1^ii^	0.90	2.49	3.1915 (17)	136
C6—H6⋯Cl1^i^	0.94	2.82	3.6323 (16)	145

Symmetry codes: (i) 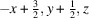 ; (ii) 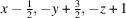 ; (iii) 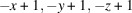 .

**Table 2:** Hydrogen-bond geometry (Å, °) for **I** at 100 K[Chem scheme1]

*D*—H⋯*A*	*D*—H	H⋯*A*	*D*⋯*A*	*D*—H⋯*A*
N1—H11*N*⋯Cl2	0.880 (15)	2.279 (15)	3.1450 (8)	167.7 (13)
N1—H12*N*⋯Cl1^i^	0.870 (14)	2.573 (14)	3.2480 (7)	135.2 (13)
N1—H12*N*⋯Cl2^ii^	0.870 (14)	2.735 (15)	3.3797 (8)	132.1 (12)
N1—H13*N*⋯Cl1	0.926 (15)	2.304 (16)	3.2080 (8)	165.0 (13)
N2—H21*N*⋯Cl1^iii^	0.901 (14)	2.487 (15)	3.2318 (8)	140.3 (12)
N2—H21*N*⋯Cl2^iv^	0.901 (14)	2.795 (14)	3.4047 (8)	126.2 (11)
N2—H22*N*⋯Cl1	0.899 (14)	2.300 (15)	3.1916 (8)	171.3 (12)
N2—H23*N*⋯Cl2	0.907 (15)	2.234 (15)	3.1201 (8)	165.5 (13)
C2—H2⋯Cl1	0.95	2.98	3.7487 (8)	139
C6—H6⋯Cl2^ii^	0.95	2.77	3.6055 (8)	147

Symmetry codes: (i) 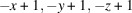 ; (ii) 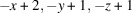 ; (iii) 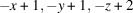 ; (iv) 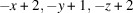 .

**Table 3:** Experimental details

	**I** at 250 K	**I** at 100 K
Crystal data		
Chemical formula	C_7_H_10_NO^+^·Cl^−^	C_7_H_10_NO^+^·Cl^−^
*M*_r_	159.61	159.61
Crystal system, space group	Orthorhombic, *P* *b* *c* *a*	Monoclinic, *P*2_1_/*c*
Temperature (K)	250	100
*a*, *b*, *c* (Å)	8.8689 (4), 8.4361 (3), 21.7319 (9)	8.3039 (6), 21.6993 (15), 8.8495 (6)
α, β, γ (°)	90, 90, 90	90, 90.077 (2), 90
*V* (Å^3^)	1625.96 (12)	1594.58 (19)
*Z*	8	8
Radiation type	Mo *K*α	Mo *K*α
μ (mm^−1^)	0.40	0.41
Crystal size (mm)	0.34 × 0.31 × 0.15	0.43 × 0.41 × 0.21

Data collection		
Diffractometer	Bruker Kappa APEXII CCD	Bruker Kappa APEXII CCD
Absorption correction	Multi-scan (*SADABS*; Krause *et al.*, 2015 ▸)	Multi-scan (*SADABS*; Krause *et al.*, 2015 ▸)
*T*_min_, *T*_max_	0.896, 0.942	0.873, 0.919
No. of measured, independent and observed [*I* > 2σ(*I*)] reflections	73425, 2717, 2019	48397, 10832, 9310
*R*_int_	0.050	0.033
(sin θ/λ)_max_ (Å^−1^)	0.737	0.940

Refinement		
*R*[*F* ^2^ > 2σ(*F* ^2^)], *wR*(*F* ^2^), *S*	0.041, 0.102, 1.08	0.033, 0.075, 1.06
No. of reflections	2717	10832
No. of parameters	93	202
H-atom treatment	H-atom parameters constrained	H atoms treated by a mixture of independent and constrained refinement
Δρ_max_, Δρ_min_ (e Å^−3^)	0.24, −0.22	0.47, −0.28

Computer programs: *APEX3* and *SAINT* (Bruker, 2016 ▸), *SHELXT2018/2* (Sheldrick, 2015 ▸), *SHELXL2018/1* (Sheldrick, 2015 ▸), *Mercury* (Macrae *et al.*, 2020 ▸), and *publCIF* (Westrip, 2010 ▸).

## supplementary crystallographic information

### 4-Methoxyanilinium chloride (I_250K) Crystal data

**Table:**

C_7_H_10_NO^+^·Cl^−^	*D*_x_ = 1.304 Mg m^−^^3^
*M**_r_* = 159.61	Mo *K*α radiation, λ = 0.71073 Å
Orthorhombic, *P**b**c**a*	Cell parameters from 9997 reflections
*a* = 8.8689 (4) Å	θ = 3.8–27.4°
*b* = 8.4361 (3) Å	µ = 0.40 mm^−^^1^
*c* = 21.7319 (9) Å	*T* = 250 K
*V* = 1625.96 (12) Å^3^	Lath fragment, pink
*Z* = 8	0.34 × 0.31 × 0.15 mm
*F*(000) = 672	

### 4-Methoxyanilinium chloride (I_250K) Data collection

**Table:**

Bruker Kappa APEXII CCD diffractometer	2717 independent reflections
Radiation source: fine-focus sealed tube	2019 reflections with *I* > 2σ(*I*)
TRIUMPH curved graphite monochromator	*R*_int_ = 0.050
φ and ω scans	θ_max_ = 31.6°, θ_min_ = 1.9°
Absorption correction: multi-scan (SADABS; Krause *et al.*, 2015)	*h* = −13→13
*T*_min_ = 0.896, *T*_max_ = 0.942	*k* = −12→12
73425 measured reflections	*l* = −32→32

### 4-Methoxyanilinium chloride (I_250K) Refinement

**Table:**

Refinement on *F*^2^	Hydrogen site location: inferred from neighbouring sites
Least-squares matrix: full	H-atom parameters constrained
*R*[*F*^2^ > 2σ(*F*^2^)] = 0.041	*w* = 1/[σ^2^(*F*_o_^2^) + (0.0348*P*)^2^ + 0.6343*P*] where *P* = (*F*_o_^2^ + 2*F*_c_^2^)/3
*wR*(*F*^2^) = 0.102	(Δ/σ)_max_ = 0.001
*S* = 1.08	Δρ_max_ = 0.24 e Å^−^^3^
2717 reflections	Δρ_min_ = −0.21 e Å^−^^3^
93 parameters	Extinction correction: SHELXL-2017/1 (Sheldrick 2015), Fc^*^=kFc[1+0.001xFc^2^λ^3^/sin(2θ)]^-1/4^
0 restraints	Extinction coefficient: 0.0070 (9)

### 4-Methoxyanilinium chloride (I_250K) Special details

**Table:**

Geometry. All esds (except the esd in the dihedral angle between two l.s. planes) are estimated using the full covariance matrix. The cell esds are taken into account individually in the estimation of esds in distances, angles and torsion angles; correlations between esds in cell parameters are only used when they are defined by crystal symmetry. An approximate (isotropic) treatment of cell esds is used for estimating esds involving l.s. planes.

### 4-Methoxyanilinium chloride (I_250K) Fractional atomic coordinates and isotropic or equivalent isotropic displacement parameters (Å^2^)

**Table:**

	*x*	*y*	*z*	*U*_iso_*/*U*_eq_	Occ. (<1)
Cl1	0.74847 (4)	0.51434 (4)	0.52125 (2)	0.03992 (12)	
O1	0.65326 (14)	0.80202 (15)	0.20491 (5)	0.0514 (3)	
N1	0.52441 (19)	0.75043 (18)	0.45535 (6)	0.0547 (4)	
H1N	0.584233	0.818422	0.475901	0.082*	0.5
H2N	0.427238	0.776832	0.461481	0.082*	0.5
H3N	0.540574	0.651270	0.469129	0.082*	0.5
H4N	0.450464	0.679260	0.461773	0.082*	0.5
H5N	0.607459	0.720851	0.476193	0.082*	0.5
H6N	0.494123	0.846412	0.468545	0.082*	0.5
C1	0.55894 (17)	0.75820 (17)	0.38936 (7)	0.0388 (3)	
C2	0.48090 (17)	0.66401 (19)	0.34891 (7)	0.0427 (3)	
H2	0.408003	0.592462	0.363560	0.051*	
C3	0.51002 (17)	0.67476 (19)	0.28628 (7)	0.0406 (3)	
H3	0.456473	0.610978	0.258349	0.049*	
C4	0.61767 (17)	0.77925 (17)	0.26521 (6)	0.0371 (3)	
C5	0.6976 (2)	0.8710 (2)	0.30673 (8)	0.0481 (4)	
H5	0.772702	0.940379	0.292416	0.058*	
C6	0.6681 (2)	0.8615 (2)	0.36875 (7)	0.0484 (4)	
H6	0.721776	0.924909	0.396768	0.058*	
C7	0.5863 (2)	0.6992 (3)	0.16061 (8)	0.0638 (5)	
H7A	0.611485	0.590164	0.170382	0.096*	
H7B	0.624205	0.725123	0.119973	0.096*	
H7C	0.477664	0.712315	0.161343	0.096*	

### 4-Methoxyanilinium chloride (I_250K) Atomic displacement parameters (Å^2^)

**Table:**

	*U*^11^	*U*^22^	*U*^33^	*U*^12^	*U*^13^	*U*^23^
Cl1	0.03990 (18)	0.04090 (19)	0.03894 (18)	−0.00135 (14)	−0.00086 (16)	0.00395 (13)
O1	0.0576 (7)	0.0614 (7)	0.0351 (6)	0.0028 (6)	0.0091 (5)	0.0037 (5)
N1	0.0653 (9)	0.0622 (9)	0.0364 (7)	0.0194 (7)	0.0085 (6)	0.0064 (6)
C1	0.0407 (7)	0.0429 (7)	0.0328 (6)	0.0138 (6)	0.0038 (6)	0.0053 (6)
C2	0.0373 (7)	0.0436 (8)	0.0473 (8)	−0.0006 (6)	0.0065 (6)	0.0055 (6)
C3	0.0375 (8)	0.0435 (8)	0.0407 (7)	0.0009 (6)	0.0001 (6)	−0.0035 (6)
C4	0.0375 (7)	0.0391 (7)	0.0347 (7)	0.0073 (6)	0.0040 (6)	0.0036 (5)
C5	0.0484 (9)	0.0496 (9)	0.0463 (9)	−0.0129 (7)	0.0036 (7)	0.0052 (7)
C6	0.0528 (9)	0.0504 (9)	0.0419 (8)	−0.0056 (7)	−0.0049 (7)	−0.0028 (7)
C7	0.0643 (12)	0.0896 (14)	0.0374 (8)	0.0102 (11)	0.0031 (8)	−0.0106 (9)

### 4-Methoxyanilinium chloride (I_250K) Geometric parameters (Å, º)

**Table:**

O1—C4	1.3615 (17)	C2—C3	1.388 (2)
O1—C7	1.425 (2)	C2—H2	0.9400
N1—C1	1.4679 (19)	C3—C4	1.378 (2)
N1—H1N	0.9000	C3—H3	0.9400
N1—H2N	0.9000	C4—C5	1.384 (2)
N1—H3N	0.9000	C5—C6	1.375 (2)
N1—H4N	0.9000	C5—H5	0.9400
N1—H5N	0.9000	C6—H6	0.9400
N1—H6N	0.9000	C7—H7A	0.9700
C1—C2	1.372 (2)	C7—H7B	0.9700
C1—C6	1.377 (2)	C7—H7C	0.9700
			
C4—O1—C7	117.88 (14)	C4—C3—C2	119.77 (14)
C1—N1—H1N	109.5	C4—C3—H3	120.1
C1—N1—H2N	109.5	C2—C3—H3	120.1
H1N—N1—H2N	109.5	O1—C4—C3	124.81 (14)
C1—N1—H3N	109.5	O1—C4—C5	115.46 (14)
H1N—N1—H3N	109.5	C3—C4—C5	119.73 (13)
H2N—N1—H3N	109.5	C6—C5—C4	120.60 (15)
C1—N1—H4N	109.5	C6—C5—H5	119.7
C1—N1—H5N	109.5	C4—C5—H5	119.7
H4N—N1—H5N	109.5	C5—C6—C1	119.28 (15)
C1—N1—H6N	109.5	C5—C6—H6	120.4
H4N—N1—H6N	109.5	C1—C6—H6	120.4
H5N—N1—H6N	109.5	O1—C7—H7A	109.5
C2—C1—C6	120.83 (14)	O1—C7—H7B	109.5
C2—C1—N1	119.65 (14)	H7A—C7—H7B	109.5
C6—C1—N1	119.51 (15)	O1—C7—H7C	109.5
C1—C2—C3	119.76 (14)	H7A—C7—H7C	109.5
C1—C2—H2	120.1	H7B—C7—H7C	109.5
C3—C2—H2	120.1		
			
C6—C1—C2—C3	−1.2 (2)	C2—C3—C4—C5	1.0 (2)
N1—C1—C2—C3	177.97 (14)	O1—C4—C5—C6	178.05 (15)
C1—C2—C3—C4	0.4 (2)	C3—C4—C5—C6	−1.5 (2)
C7—O1—C4—C3	−7.0 (2)	C4—C5—C6—C1	0.7 (3)
C7—O1—C4—C5	173.48 (15)	C2—C1—C6—C5	0.7 (2)
C2—C3—C4—O1	−178.58 (14)	N1—C1—C6—C5	−178.52 (15)

### 4-Methoxyanilinium chloride (I_250K) Hydrogen-bond geometry (Å, º)

**Table:**

*D*—H···*A*	*D*—H	H···*A*	*D*···*A*	*D*—H···*A*
N1—H1*N*···Cl1^i^	0.90	2.43	3.3265 (18)	174
N1—H2*N*···Cl1^ii^	0.90	2.40	3.1915 (15)	147
N1—H3*N*···CL1	0.90	2.45	3.1570 (16)	135
N1—H4*N*···Cl1^iii^	0.90	2.43	3.3323 (18)	180
N1—H5*N*···Cl1	0.90	2.36	3.1570 (15)	148
N1—H6*N*···Cl1^ii^	0.90	2.49	3.1915 (17)	136
C6—H6···Cl1^i^	0.94	2.82	3.6323 (16)	145

Symmetry codes: (i) −*x*+3/2, *y*+1/2, *z*; (ii) *x*−1/2, −*y*+3/2, −*z*+1; (iii) −*x*+1, −*y*+1, −*z*+1.

### 4-Methoxyanilinium chloride (I_100K) Crystal data

**Table:**

C_7_H_10_NO^+^·Cl^−^	*F*(000) = 672
*M**_r_* = 159.61	*D*_x_ = 1.330 Mg m^−^^3^
Monoclinic, *P*2_1_/*c*	Mo *K*α radiation, λ = 0.71073 Å
*a* = 8.3039 (6) Å	Cell parameters from 9875 reflections
*b* = 21.6993 (15) Å	θ = 3.4–41.5°
*c* = 8.8495 (6) Å	µ = 0.41 mm^−^^1^
β = 90.077 (2)°	*T* = 100 K
*V* = 1594.58 (19) Å^3^	Needle fragment, pink
*Z* = 8	0.43 × 0.41 × 0.21 mm

### 4-Methoxyanilinium chloride (I_100K) Data collection

**Table:**

Bruker Kappa APEXII CCD diffractometer	10832 independent reflections
Radiation source: fine-focus sealed tube	9310 reflections with *I* > 2σ(*I*)
TRIUMPH curved graphite monochromator	*R*_int_ = 0.033
φ and ω scans	θ_max_ = 41.9°, θ_min_ = 1.9°
Absorption correction: multi-scan (SADABS; Krause *et al.*, 2015)	*h* = −15→15
*T*_min_ = 0.873, *T*_max_ = 0.919	*k* = −40→40
48397 measured reflections	*l* = −16→15

### 4-Methoxyanilinium chloride (I_100K) Refinement

**Table:**

Refinement on *F*^2^	Primary atom site location: dual
Least-squares matrix: full	Hydrogen site location: mixed
*R*[*F*^2^ > 2σ(*F*^2^)] = 0.033	H atoms treated by a mixture of independent and constrained refinement
*wR*(*F*^2^) = 0.075	*w* = 1/[σ^2^(*F*_o_^2^) + (0.0336*P*)^2^ + 0.1702*P*] where *P* = (*F*_o_^2^ + 2*F*_c_^2^)/3
*S* = 1.06	(Δ/σ)_max_ = 0.001
10832 reflections	Δρ_max_ = 0.47 e Å^−^^3^
202 parameters	Δρ_min_ = −0.28 e Å^−^^3^
0 restraints	

### 4-Methoxyanilinium chloride (I_100K) Special details

**Table:**

Geometry. All esds (except the esd in the dihedral angle between two l.s. planes) are estimated using the full covariance matrix. The cell esds are taken into account individually in the estimation of esds in distances, angles and torsion angles; correlations between esds in cell parameters are only used when they are defined by crystal symmetry. An approximate (isotropic) treatment of cell esds is used for estimating esds involving l.s. planes.
Refinement. Refined as a 2-component twin

### 4-Methoxyanilinium chloride (I_100K) Fractional atomic coordinates and isotropic or equivalent isotropic displacement parameters (Å^2^)

**Table:**

	*x*	*y*	*z*	*U*_iso_*/*U*_eq_	
Cl1	0.46961 (2)	0.52412 (2)	0.74414 (2)	0.01433 (3)	
Cl2	1.00704 (2)	0.51981 (2)	0.75416 (2)	0.01431 (3)	
O1	0.81503 (9)	0.79863 (3)	0.36295 (8)	0.01972 (12)	
N1	0.75005 (10)	0.54837 (3)	0.50236 (8)	0.01524 (11)	
H11N	0.8313 (18)	0.5382 (6)	0.5613 (16)	0.023*	
H12N	0.7496 (18)	0.5254 (6)	0.4219 (16)	0.023*	
H13N	0.6580 (18)	0.5390 (6)	0.5566 (17)	0.023*	
C1	0.75955 (9)	0.61391 (3)	0.46333 (8)	0.01282 (11)	
C2	0.66656 (10)	0.65590 (4)	0.54219 (9)	0.01463 (13)	
H2	0.592916	0.642048	0.616899	0.018*	
C3	0.68159 (10)	0.71877 (4)	0.51130 (9)	0.01510 (12)	
H3	0.618985	0.747903	0.565734	0.018*	
C4	0.78835 (10)	0.73857 (3)	0.40075 (9)	0.01439 (12)	
C5	0.87901 (11)	0.69542 (4)	0.31922 (10)	0.01746 (14)	
H5	0.949889	0.708935	0.241833	0.021*	
C6	0.86556 (10)	0.63310 (4)	0.35115 (10)	0.01644 (13)	
H6	0.928009	0.603820	0.297051	0.020*	
C7	0.71299 (15)	0.84409 (4)	0.43121 (12)	0.02472 (19)	
H7A	0.600193	0.834617	0.408214	0.037*	
H7B	0.739987	0.884893	0.391123	0.037*	
H7C	0.729058	0.843809	0.540940	0.037*	
O2	0.69262 (8)	0.20673 (3)	0.82982 (8)	0.01905 (11)	
N2	0.73702 (9)	0.45944 (3)	0.94929 (8)	0.01440 (11)	
H21N	0.7316 (18)	0.4677 (6)	1.0489 (16)	0.022*	
H22N	0.6576 (18)	0.4795 (6)	0.9008 (16)	0.022*	
H23N	0.8253 (18)	0.4776 (6)	0.9085 (16)	0.022*	
C8	0.72962 (9)	0.39304 (3)	0.91915 (8)	0.01249 (11)	
C9	0.82458 (10)	0.35313 (4)	1.00292 (9)	0.01498 (12)	
H9	0.895113	0.368738	1.078368	0.018*	
C10	0.81599 (10)	0.28992 (3)	0.97583 (9)	0.01480 (12)	
H10	0.880248	0.262235	1.033186	0.018*	
C11	0.71267 (9)	0.26745 (3)	0.86417 (9)	0.01383 (12)	
C12	0.61959 (10)	0.30835 (4)	0.77920 (10)	0.01777 (14)	
H12	0.550715	0.293062	0.702026	0.021*	
C13	0.62716 (10)	0.37124 (4)	0.80690 (10)	0.01671 (13)	
H13	0.563053	0.399067	0.749807	0.020*	
C14	0.80131 (12)	0.16348 (4)	0.89757 (11)	0.02172 (16)	
H14A	0.912339	0.174734	0.872250	0.033*	
H14B	0.778211	0.122036	0.859247	0.033*	
H14C	0.787721	0.164090	1.007547	0.033*	

### 4-Methoxyanilinium chloride (I_100K) Atomic displacement parameters (Å^2^)

**Table:**

	*U*^11^	*U*^22^	*U*^33^	*U*^12^	*U*^13^	*U*^23^
Cl1	0.01254 (6)	0.01532 (6)	0.01511 (7)	−0.00041 (5)	−0.00015 (8)	0.00016 (6)
Cl2	0.01256 (6)	0.01453 (6)	0.01584 (7)	−0.00127 (5)	−0.00072 (8)	−0.00022 (6)
O1	0.0248 (3)	0.0133 (2)	0.0211 (3)	−0.0012 (2)	−0.0017 (2)	0.0031 (2)
N1	0.0200 (3)	0.0126 (2)	0.0132 (3)	−0.0018 (2)	−0.0019 (2)	0.00002 (19)
C1	0.0144 (3)	0.0126 (2)	0.0114 (3)	−0.0006 (2)	−0.0012 (2)	−0.0003 (2)
C2	0.0154 (3)	0.0155 (3)	0.0130 (3)	−0.0001 (2)	0.0007 (2)	−0.0002 (2)
C3	0.0168 (3)	0.0149 (3)	0.0136 (3)	0.0019 (2)	−0.0001 (2)	−0.0010 (2)
C4	0.0158 (3)	0.0133 (3)	0.0140 (3)	−0.0011 (2)	−0.0034 (2)	0.0009 (2)
C5	0.0187 (3)	0.0169 (3)	0.0168 (3)	−0.0015 (3)	0.0047 (3)	0.0013 (3)
C6	0.0182 (3)	0.0150 (3)	0.0161 (3)	0.0007 (2)	0.0037 (3)	−0.0010 (2)
C7	0.0372 (6)	0.0143 (3)	0.0226 (4)	0.0052 (3)	−0.0034 (4)	0.0003 (3)
O2	0.0220 (3)	0.0129 (2)	0.0222 (3)	−0.0016 (2)	−0.0008 (2)	−0.0029 (2)
N2	0.0182 (3)	0.0126 (2)	0.0124 (3)	−0.0008 (2)	0.0008 (2)	0.00002 (19)
C8	0.0136 (3)	0.0124 (2)	0.0115 (3)	−0.0012 (2)	0.0008 (2)	−0.0003 (2)
C9	0.0166 (3)	0.0149 (3)	0.0134 (3)	−0.0003 (2)	−0.0026 (3)	−0.0014 (2)
C10	0.0169 (3)	0.0139 (3)	0.0136 (3)	0.0007 (2)	−0.0013 (2)	−0.0006 (2)
C11	0.0147 (3)	0.0129 (3)	0.0139 (3)	−0.0023 (2)	0.0023 (2)	−0.0014 (2)
C12	0.0193 (3)	0.0160 (3)	0.0180 (4)	−0.0021 (2)	−0.0055 (3)	−0.0018 (2)
C13	0.0181 (3)	0.0153 (3)	0.0167 (3)	−0.0010 (2)	−0.0050 (3)	0.0011 (2)
C14	0.0280 (4)	0.0136 (3)	0.0236 (4)	0.0022 (3)	0.0032 (3)	−0.0011 (3)

### 4-Methoxyanilinium chloride (I_100K) Geometric parameters (Å, º)

**Table:**

O1—C4	1.3636 (10)	O2—C11	1.3624 (9)
O1—C7	1.4343 (12)	O2—C14	1.4330 (11)
N1—C1	1.4655 (10)	N2—C8	1.4667 (10)
N1—H11N	0.880 (15)	N2—H21N	0.901 (14)
N1—H12N	0.870 (14)	N2—H22N	0.899 (14)
N1—H13N	0.926 (15)	N2—H23N	0.907 (15)
C1—C2	1.3838 (11)	C8—C9	1.3856 (11)
C1—C6	1.3915 (11)	C8—C13	1.3900 (11)
C2—C3	1.3971 (11)	C9—C10	1.3941 (11)
C2—H2	0.9500	C9—H9	0.9500
C3—C4	1.3893 (12)	C10—C11	1.3957 (11)
C3—H3	0.9500	C10—H10	0.9500
C4—C5	1.4020 (12)	C11—C12	1.3961 (11)
C5—C6	1.3860 (11)	C12—C13	1.3879 (11)
C5—H5	0.9500	C12—H12	0.9500
C6—H6	0.9500	C13—H13	0.9500
C7—H7A	0.9800	C14—H14A	0.9800
C7—H7B	0.9800	C14—H14B	0.9800
C7—H7C	0.9800	C14—H14C	0.9800
			
C4—O1—C7	117.24 (7)	C11—O2—C14	117.60 (7)
C1—N1—H11N	109.9 (9)	C8—N2—H21N	111.7 (8)
C1—N1—H12N	111.4 (9)	C8—N2—H22N	111.0 (8)
H11N—N1—H12N	110.1 (13)	H21N—N2—H22N	109.5 (12)
C1—N1—H13N	112.4 (8)	C8—N2—H23N	112.8 (8)
H11N—N1—H13N	105.7 (12)	H21N—N2—H23N	110.2 (13)
H12N—N1—H13N	107.2 (13)	H22N—N2—H23N	101.1 (12)
C2—C1—C6	121.11 (7)	C9—C8—C13	121.13 (7)
C2—C1—N1	119.33 (7)	C9—C8—N2	119.54 (7)
C6—C1—N1	119.53 (7)	C13—C8—N2	119.32 (7)
C1—C2—C3	119.63 (8)	C8—C9—C10	119.60 (7)
C1—C2—H2	120.2	C8—C9—H9	120.2
C3—C2—H2	120.2	C10—C9—H9	120.2
C4—C3—C2	119.81 (7)	C9—C10—C11	119.78 (7)
C4—C3—H3	120.1	C9—C10—H10	120.1
C2—C3—H3	120.1	C11—C10—H10	120.1
O1—C4—C3	124.91 (7)	O2—C11—C10	124.79 (7)
O1—C4—C5	115.12 (7)	O2—C11—C12	115.29 (7)
C3—C4—C5	119.96 (7)	C10—C11—C12	119.91 (7)
C6—C5—C4	120.22 (8)	C13—C12—C11	120.33 (7)
C6—C5—H5	119.9	C13—C12—H12	119.8
C4—C5—H5	119.9	C11—C12—H12	119.8
C5—C6—C1	119.26 (7)	C12—C13—C8	119.23 (7)
C5—C6—H6	120.4	C12—C13—H13	120.4
C1—C6—H6	120.4	C8—C13—H13	120.4
O1—C7—H7A	109.5	O2—C14—H14A	109.5
O1—C7—H7B	109.5	O2—C14—H14B	109.5
H7A—C7—H7B	109.5	H14A—C14—H14B	109.5
O1—C7—H7C	109.5	O2—C14—H14C	109.5
H7A—C7—H7C	109.5	H14A—C14—H14C	109.5
H7B—C7—H7C	109.5	H14B—C14—H14C	109.5
			
C6—C1—C2—C3	−1.43 (12)	C13—C8—C9—C10	−0.84 (12)
N1—C1—C2—C3	176.47 (7)	N2—C8—C9—C10	179.11 (7)
C1—C2—C3—C4	0.67 (12)	C8—C9—C10—C11	0.36 (12)
C7—O1—C4—C3	−6.94 (12)	C14—O2—C11—C10	−9.35 (12)
C7—O1—C4—C5	173.41 (8)	C14—O2—C11—C12	171.16 (8)
C2—C3—C4—O1	−178.79 (8)	C9—C10—C11—O2	−178.87 (8)
C2—C3—C4—C5	0.84 (12)	C9—C10—C11—C12	0.60 (12)
O1—C4—C5—C6	178.02 (8)	O2—C11—C12—C13	178.42 (8)
C3—C4—C5—C6	−1.65 (12)	C10—C11—C12—C13	−1.10 (13)
C4—C5—C6—C1	0.91 (13)	C11—C12—C13—C8	0.63 (13)
C2—C1—C6—C5	0.63 (12)	C9—C8—C13—C12	0.35 (13)
N1—C1—C6—C5	−177.26 (8)	N2—C8—C13—C12	−179.60 (8)

### 4-Methoxyanilinium chloride (I_100K) Hydrogen-bond geometry (Å, º)

**Table:**

*D*—H···*A*	*D*—H	H···*A*	*D*···*A*	*D*—H···*A*
N1—H11*N*···Cl2	0.880 (15)	2.279 (15)	3.1450 (8)	167.7 (13)
N1—H12*N*···Cl1^i^	0.870 (14)	2.573 (14)	3.2480 (7)	135.2 (13)
N1—H12*N*···Cl2^ii^	0.870 (14)	2.735 (15)	3.3797 (8)	132.1 (12)
N1—H13*N*···Cl1	0.926 (15)	2.304 (16)	3.2080 (8)	165.0 (13)
N2—H21*N*···Cl1^iii^	0.901 (14)	2.487 (15)	3.2318 (8)	140.3 (12)
N2—H21*N*···Cl2^iv^	0.901 (14)	2.795 (14)	3.4047 (8)	126.2 (11)
N2—H22*N*···Cl1	0.899 (14)	2.300 (15)	3.1916 (8)	171.3 (12)
N2—H23*N*···Cl2	0.907 (15)	2.234 (15)	3.1201 (8)	165.5 (13)
C2—H2···Cl1	0.95	2.98	3.7487 (8)	139
C6—H6···Cl2^ii^	0.95	2.77	3.6055 (8)	147

Symmetry codes: (i) −*x*+1, −*y*+1, −*z*+1; (ii) −*x*+2, −*y*+1, −*z*+1; (iii) −*x*+1, −*y*+1, −*z*+2; (iv) −*x*+2, −*y*+1, −*z*+2.
